# Evaluating Novel Braided Metal Stent for Bilateral Simultaneous Side-by-Side Stenting in Malignant Hilar Biliary Obstruction: A Multicenter, Single-Arm Prospective Study

**DOI:** 10.3390/jcm14186557

**Published:** 2025-09-18

**Authors:** Jungnam Lee, Seok Jeong, Eui Joo Kim, Huapyong Kang, Dong Uk Kim, Chang-Il Kwon

**Affiliations:** 1 Division of Gastroenterology, Department of Internal Medicine, Inha University Hospital, Inha University College of Medicine, Incheon 22332, Republic of Korea; jungnamlee801105@inha.ac.kr; 2 Department of Internal Medicine, Gil Medical Center, Gachon University College of Medicine, Incheon 21565, Republic of Korea; imetkim@gilhospital.com (E.J.K.); rabbit9644@gmail.com (H.K.); 3 Department of Internal Medicine, CHA University Gumi Medical Center, Gumi 39295, Republic of Korea; amlm3@hanmail.net; 4 Digestive Disease Center, Department of Internal Medicine, CHA Bundang Medical Center, CHA University, Seongnam 13496, Republic of Korea; mdkwon@naver.com

**Keywords:** self-expandable metal stent, malignant hilar biliary obstruction, simultaneous side-by-side stent placement, stent patency

## Abstract

**Background**: While the side-by-side stenting technique-characterized by the parallel placement of stents offers procedural simplicity, the augmented radial force exerted by the initial stent may complicate subsequent deployment. This multicenter study evaluated the practicality and safety of bilateral side-by-side stenting using novel braided self-expandable metal stents (Benefit^TM^; M.I.Tech Co., Ltd., Pyeongtaek, Republic of Korea). Statistical analysis included survival analysis (Kaplan–Meier) and Cox proportional hazards regression to identify predictive factors. **Patients and Methods**: In this multicenter study, patients with inoperable malignant hilar biliary obstruction (Bismuth type II–IV) underwent simultaneous side-by-side endoscopic placement of two braided self-expandable metal stents. The primary outcome was stent patency. The secondary outcomes included technical and clinical success, and adverse events monitored for up to one year. **Results**: A total of 27 patients were included in the final analysis. The technical success rate was 92.6% (25/27), and the clinical success rate was 88.0% (22/25). The median stent patency was 93 days, with cumulative patency rates of 87.4% at 3 months and 49.7% at 12 months. Tumor ingrowth was the most common cause of stent occlusion (66.7%). Early adverse events occurred in 2 patients (one cholangitis and one stent migration), supporting the favorable safety profile of this approach. **Conclusions**: The simultaneous side-by-side placement of novel braided self-expandable metal stents yielded high technical success and favorable clinical outcomes in patients with inoperable malignant hilar biliary obstruction. This approach provided substantial stent patency with a low complication rate, supporting its utility as a safe and effective palliative strategy for the management of malignant hilar biliary obstruction.

## 1. Introduction

Malignant hilar biliary obstruction (MHBO) is a complex clinical condition resulting from compression of the hepatic hilum, most commonly by hilar cholangiocarcinoma (Klatskin tumor) and gallbladder cancer, but also by other primary or metastatic malignancies [1,2]. Notably, curative surgical options for MHBO are limited as patients often present with an advanced stage of disease at the time of diagnosis, thereby necessitating palliative management [1].

Restoring effective biliary drainage to alleviate severe complications resulting from obstruction is the primary focus of treatment in patients with unresectable obstruction. Effective drainage relieves cholestasis and reduces the risk of complications such as obstructive jaundice, recurrent cholangitis, and hepatic dysfunction, which have a profound impact on the quality of life and overall prognosis [1,3]. Given the longer duration of patency and decreased requirement for reintervention achieved with their use, self-expandable metal stents (SEMS) have become the cornerstone of palliative treatment. The use of SEMS has enabled patients to better tolerate adjunctive therapies [4,5].

The optimal stenting technique for MHBO remains controversial despite the advantages of SEMS. Bilateral stenting facilitates the drainage of a larger proportion of the liver, thereby aiding in the prevention of jaundice. However, it also presents significant technical challenges [6,7,8] such as limited space within the common bile duct, difficulty in achieving precise stent alignment, and the risk of stent compression or overlap. These challenges have led to the technical success rates of bilateral deployment being lower than those of unilateral stenting. Several studies have compared various deployment approaches, such as the stent-in-stent and side-by-side techniques and elucidated their advantages and limitations [4,9].

This prospective pilot study aimed to evaluate the efficacy of simultaneous side-by-side bilateral deployment of a newly developed braided SEMS using a dedicated 5.9 Fr introducer. The primary objective of the present study was to determine whether this innovative technique could achieve high technical and clinical success, prolong the duration of stent patency, and offer safe and effective palliative treatment for patients with unresectable MHBO.

## 2. Patients and Methods

### 2.1. Study Design and Patients

This was a prospective, single-arm, multicenter, pilot trial that included individuals who underwent bilateral insertion of self-expandable metal stents (SEMS; Benefit^TM^, M. I. Tech Co., Ltd., Pyeongtaek, Republic of Korea) using a side-by-side technique between February 2020 and March 2023 across four tertiary care centers in Korea (Inha University Hospital, Gil Medical Center, CHA Medical Center, and Pusan National University Hospital). Multidisciplinary discussions involving surgeons and oncologists were conducted pre-operatively to evaluate the potential for surgical resection. Candidates for bilateral stenting were identified based on factors such as tumor extent, intrahepatic duct dilatation, and residual liver volume. The initial management of patients presenting with cholangitis upon admission comprised nasobiliary drainage; stent placement was deferred until clinical stabilization was achieved.

Patients who met the following criteria were included in the study: (1) age ≥ 19 years, (2) a diagnosis of unresectable MHBO confirmed through pathological or clinical assessment, (3) hilar tumors of Bismuth type II, III, or IV, and (4) an estimated survival expectancy of >3 months. Participants who met any of the following criteria were excluded from this study: (1) contraindications to endoscopic retrograde cholangiopancreatography (ERCP); (2) uncorrected coagulopathy, defined as a prothrombin time-international normalized ratio (PT-INR) of >1.5 or platelet count of <60,000/mm^3^; (3) a history of surgery that significantly alters the upper gastrointestinal or biliary anatomy (e.g., gastrectomy with Billroth II or Roux-en-Y reconstruction, or a biliary-enteric anastomosis such as choledochojejunostomy), excluding cholecystectomy; (4) SEMS placement in the biliary system; and (5) the requirement for simultaneous percutaneous biliary drainage in addition to endoscopic stenting.

### 2.2. Benefit^TM^ Biliary SEMSs

**Figure 1:**
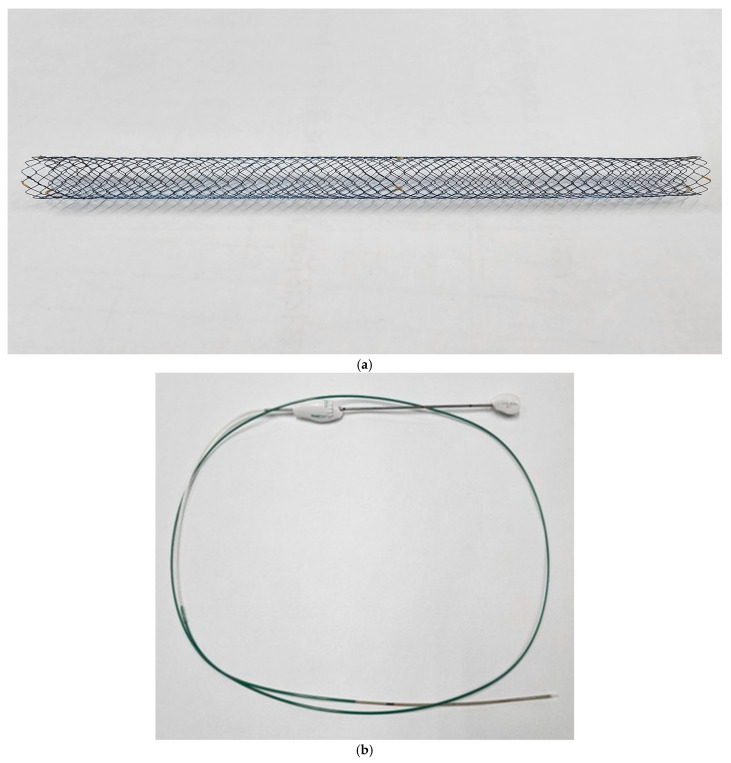
(**a**) The uncovered biliary braided metal stents together with (**b**) a 5.9 Fr introducer (Benefit™) designed for the simultaneous, side-by-side deployment of two stents in the peri-hilar bile ducts.

Uncovered biliary braided metal stents were used along with a 5.9 Fr introducer (Benefit™) designed for the simultaneous, side-by-side deployment of two stents in the peri-hilar bile ducts in the present study. This ultrathin introducer decreases friction between the introducers of SEMSs within the bile duct and the working channel of the duodenoscope, thereby enhancing the ability to navigate through the narrowed perihilar bile ducts and ensuring optimal stent placement. Each stent measured 8 mm in diameter and was 6, 8, 10, or 12 cm long, with a shortening rate of 20% (Figure 1a,b).

### 2.3. ERCP-Guided Simultaneous Bilateral SEMS Placement Using the Side-by-Side Technique

Experienced endoscopists who perform >500 endoscopic retrograde cholangiopancreatography (ERCP) procedures annually performed all procedures [10]. A duodenoscope (TJF-260V; Olympus Medical Systems Corporation, Tokyo, Japan) with fluoroscopic guidance was used during the procedure. Two 0.025-inch guidewires were navigated into the left and right hepatic ducts after achieving biliary cannulation. The guide wires were positioned in the right anterior and posterior segmental ducts if access to the left hepatic duct could not be achieved. Benefit^TM^ stents were used in all cases. The length of the stents (5, 6, 8, 10, and 12 cm) was selected based on the anatomical characteristics of the biliary stricture. Two 5.9 Fr stent introducers pre-loaded with 8 mm-diameter biliary SEMS were simultaneously advanced into the targeted intrahepatic ducts. Radiopaque markers were used to confirm proper positioning at the stricture site. Meticulous attention was paid during simultaneous deployment to ensure that the distal ends of the stents were aligned at the same level to optimize biliary drainage.

### 2.4. Outcome Measurements and Definitions

The stent patency period, defined as the duration from the placement of the biliary SEMS placement to the onset of stent dysfunction, was defined as the primary endpoint of this study. An obstruction caused by neoplastic tissue proliferation, accumulation of biliary sludge or calculi, or hemorrhage leading to biliary obstruction was defined as stent dysfunction. Occlusions occurring in anatomical regions distant from the stent, such as the distal common bile duct, were not considered stent dysfunction. Patients who died or were lost to follow-up before stent dysfunction were excluded from the analysis.

Technical success, clinical success, adverse events, risk factors for stent dysfunction, and overall survival were defined as the secondary outcomes. The accurate placement of bilateral stents using the side-by-side technique was defined as technical success. A decrease in bilirubin levels to ≤20% of the pre-treatment value, normalization (if applicable), or a reduction to ≤25% of the pre-treatment level within 1 month after the procedure was defined as clinical success. The highest recorded levels of alkaline phosphatase (ALP) and bilirubin during the hospitalization period and the procedure were also documented. The date of biopsy-confirmed malignancy was defined as the date of diagnosis. The date of diagnosis was determined based on the first radiologic evidence of biliary obstruction, onset of related symptoms (e.g., fever and jaundice), or the date of the initial biliary drainage procedure for patients with metastatic cancer. The presence of cholangitis, defined as fever with jaundice with or without right upper quadrant pain, prior to stent insertion was noted. Adverse events were classified as intra- or post-procedural. Post-procedural events were defined as those occurring within 14 days of the procedure in accordance with standardized endoscopic adverse event criteria. Overall survival (OS) was calculated from the procedure date to the date of death or last follow-up visit.

### 2.5. Statistical Methods

The data are presented as the median (range) and percentages. Kaplan–Meier survival analysis was performed to estimate the stent patency and OS over time. Univariate and multivariate Cox proportional hazards regression analyses were conducted using a stepwise selection strategy to evaluate the factors potentially affecting stent patency and likelihood ratio tests were employed to assess the categorical variables. The proportional hazards assumption verified using Schoenfeld residuals revealed no violations (*p* > 0.05). A two-sided *p*-value of <0.05 was considered statistically significant. All statistical analyses were conducted using SPSS version 19.0 (SPSS Corp., Armonk, NY, USA).

### 2.6. Ethics Approval

The Institutional Review Board (IRB) of Inha University Hospital (IRB No. INHAUH 2018-06-031) approved the study protocol. Written informed consent was obtained from all participants before their inclusion in the study. The study adhered to the principles outlined in the Declaration of Helsinki and complied with Good Clinical Practice standards. The patient data were anonymized prior to analysis to ensure privacy and confidentiality. Enrollment was limited to individuals of Korean ethnicity. This research was registered with the Clinical Research Information Service (CRIS) of the Ministry of Health and Welfare of the Republic of Korea (CRIS number: KCT0003617).

## 3. Results

### 3.1. Baseline Characteristics

**Figure 2:**
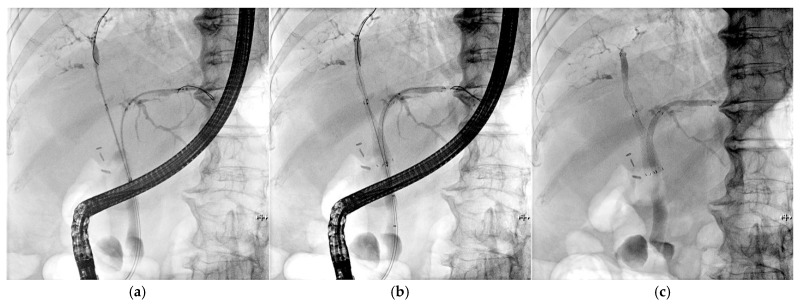
The Benefit^TM^ stent inserted in a patient with a Klatskin tumor (Bismuth IV). (**a**) A 5.9 Fr introducer inserted into the B2 and B8 branches. (**b**) Deployment of the SEMS. (**c**) Benefit stent inserted in the patient.

Among the 31 patients with malignant hilar obstruction who underwent simultaneous bilateral stenting, four patients who were transferred to other institutions for supportive care were excluded from the final analysis. Thus, 27 patients were evaluated. Pathological confirmation of malignancy was obtained in all cases, except for 2 patients wherein hilar cholangiocarcinoma was diagnosed based solely on imaging findings and clinical courses. The median age of the study cohort, comprising 16 males and 11 females, was 76 years (range, 52–92 years). The underlying malignancies were hilar cholangiocarcinoma, carcinoma of the gallbladder, and metastatic disease in 20 patients (74.1%), five (18.5%), and two (7.4%) patients, respectively. Two patients were diagnosed with colorectal cancer and hepatocellular carcinoma (*n* = 1 each). According to the Bismuth classification, 10 (37.0%), 1 (3.7%), and 16 (59.3%) lesions were types II, III, and IV, respectively. The pre-procedure laboratory tests revealed that the median alkaline phosphatase (ALP) and bilirubin levels were 328 IU/dL (range: 98–1489) and 4.9 mg/dL (range: 0.3–25.6), respectively. Post-stent placement chemotherapy was commenced in 19 of the 27 patients (70.4%). Table 1 presents the detailed baseline characteristics of the patients. Figure 2 presents the Benefit^TM^ stent inserted into the patient.

### 3.2. Clinical Outcomes and Safety

Technical and clinical success were achieved in 25 (92.6%) and 22 (88.0%) of the 27 enrolled patients, respectively. Technical failure occurred in 2 cases owing to the failure of selective biliary cannulation. However, these cases were successfully managed through percutaneous biliary drainage (Table 2). Clinical failure was observed in three patients. No additional interventions were required in these three cases. A slow improvement in the total bilirubin levels that eventually recovered was observed in 2 patients. One patient died of infection on the 24th day after the procedure. The median duration of stent patency over the 1-year observation period was 93 days (range, 9–365 days). Stent dysfunction was observed in six (24.0%) of the 25 patients owing to tumor ingrowth (*n* = 4) and sludge/stone formation (*n* = 2). Patency analysis demonstrated that the cumulative patency rates at 3, 6, and 12 months were 87.4%, 79.5%, and 49.7%, respectively. Table 3 presents the results of the survival analysis, indicating that the duration of stent patency encompassed the overall survival period, thereby supporting the clinical efficacy of the stent. Eighteen of the 25 patients (72.0%) had died by the end of the 1-year follow-up period owing to the progression of underlying malignancies (*n* = 15) and secondary infections (*n* = 3). Postprocedural adverse events occurred in 2 (8.0%) of the 25 patients. Both patients presented with cholangitis; 1 patient was treated solely with antibiotics, whereas repeat stent insertion was required in the other patient (Table 4).

### 3.3. Factors Associated with the Duration of Biliary SEMS Patency

Univariate analyses revealed no statistically significant associations between the duration of stent patency and sex, age, type of malignancy, baseline total bilirubin levels, and baseline alkaline phosphatase levels. Multivariate modeling did not reveal any independent predictors of patency. Table 5 presents a comprehensive summary of the results.

## 4. Discussion

This prospective, multicenter, pilot trial demonstrated that the simultaneous side-by-side bilateral deployment of a novel braided SEMS using a dedicated 5.9 Fr introducer is a technically feasible, safe, and effective palliative approach for the management of patients with unresectable MHBO. The present study revealed that the median duration of stent patency was 93 days with technical and clinical success rates of 92.6% and 88.0%, respectively. The incidence of adverse events was low. Notably, stent failure of any form was not observed until the end of their lives in 64% of patients, indicating that the stents provided sustained, uninterrupted biliary drainage during the critical palliative care period. This remarkable durability minimized the requirement for additional interventions and contributed to a reduction in the incidence of the complications associated with biliary obstruction, indicating a significantly positive impact on patient quality of life.

Effective biliary decompression plays a crucial role in alleviating cholestasis, reducing recurrent cholangitis, and enhancing patient comfort [1,11,12]. Given its association with enhanced patient well-being and fewer complications, endoscopic stenting via ERCP with SEMS has been favored over percutaneous methods [13,14,15,16,17]. However, an optimal stenting approach remains to be identified. The unilateral versus bilateral relationship remains controversial. Bilateral stent placement may be associated with increased survival, duration of patency, and successful drainage [5,9,18]. However, it is also associated with lower technical success rates. Naitoh et al. and Lee et al. reported that bilateral drainage is associated with increased technical complexity owing to the requirement for bilateral deployment [5,9]. However, the findings of the present study suggest that this technical complexity can be mitigated. The use of the novel 5.9 Fr introducer yielded a high technical success rate (92.6%) and favorable clinical outcomes. This result compares favorably with previous reports, which have ranged from 76.9% to 95.5% for bilateral stenting, and demonstrates favorable clinical outcomes [5,9]. This finding indicates that the challenges inherent to bilateral stenting can be effectively mitigated using optimized techniques, appropriate devices, and patient selection.

A key technical consideration is the selection of the deployment methods of side-by-side and stent-in-stent. Zhou et al. reported that although the overall technical and clinical success rates of the two techniques are comparable, the side-by-side approach is associated with a significantly shorter procedure time (mean difference of approximately −12 min) and a favorable hazard ratio for stent patency (HR 1.22, 95% CI 1.01–1.47) [19]. This efficiency may be attributed to the relative ease with which the guidewire can be negotiated when both intrahepatic ducts are accessed simultaneously. In contrast, although it offers a more physiological configuration by positioning the stents above the papilla to reduce duodenal reflux, the technically demanding nature of stent-in-stent deployment may limit its widespread use, particularly in centers without advanced expertise. The findings of the present study suggest that the side-by-side technique is a feasible approach. The optimized technique and specialized delivery system simplified the procedure while achieving good stent patency and a low rate of complications. Thus, this approach may be a practical and widely applicable option for the bilateral stenting of malignant hilar biliary obstruction.

The present study has certain limitations that must be considered. First, larger prospective randomized trials must be conducted to validate the findings of the present study, given that this was a pilot study with a relatively small sample size. Second, the high loss to follow-up rate observed in the present study, a common challenge in the palliative setting, likely contributed to shorter survival estimates. Given the advanced disease stage of the patients included in the present study, many patients may have sought palliative care at institutions other than the study centers rather than experiencing early mortality. This may have led to the underestimation of the survival duration. Lastly, patient-reported outcomes such as quality-of-life assessments and cost-effectiveness analysis were not incorporated in the present study. Future studies must aim to integrate comprehensive quality-of-life evaluations and economic analyses to fully establish the clinical and economic value of this novel stenting approach since palliative treatment for MHBO aims to prolong survival, improve patient comfort, and reduce healthcare costs.

In conclusion, the outcomes of this preliminary analysis suggest that simultaneous bilateral deployment of novel braided SEMS via the side-by-side technique offers a promising balance among technical feasibility, safety, and clinical efficacy in patients with unresectable MHBO. The maintenance of stent function until death in 64% of patients underscores the potential of this technique to result in a significant improvement in the quality of life in a palliative setting. Further studies with long-term follow-up, quality-of-life assessments, and cost-effectiveness studies, must be conducted in the future to validate these findings and refine the patient selection and procedural protocols.

## Figures and Tables

**Table 1:** Baseline Characteristics of Patients with Malignant Hilar Biliary Obstruction (*n* = 27).

Variables	Patients (*n* = 27)
Age, year *	76 (52–92)
Sex ^§^	
Male	16 (59.3%)
Female	11 (40.7%)
Diagnosis ^§^	
Hilar cholangiocarcinoma	20 (74.1%)
Carcinoma of the gallbladder	5 (18.5%)
Other cancers	2 (7.4%)
Comorbidities ^§^	
Hypertension	10 (37.0%)
Diabetes mellitus	9 (33.3%)
Liver cirrhosis	2 (7.4%)
Coronary artery obstructive disease	2 (7.4%)
Hepatitis	1 (3.7%)
Others	2 (7.4%)
Bismuth-Corlette classification ^§^	
Type II	10 (37.0%)
Type III	1 (3.7%)
Type IV	16 (59.3%)
Laboratory examination *	
WBC (/µL)	7760 (3840–17,320)
Total bilirubin (mg/dL)	4.9 (0.3–25.7)
AST (U/L)	72.0 (21.0–1331.0)
ALT (U/L)	45.0 (11.0–517.0)
ALP (U/L)	328.0 (98.0–1489.0)
Amylase (U/L)	48.5 (8.0–200.9)
Lipase (U/L)	43.0 (5.0–574.8)

Abbreviations: WBC, white blood cell; AST, aspartate aminotransferase; ALT, alanine aminotransferase; ALP, alkaline phosphatase. *, median (min-max). ^§^, *n* (%).

**Table 2:** Technical and Clinical Success and Median Stent Patency Duration.

Variables	Results
Technical success ^§^	25/27, 92.6%
Clinical success ^§^ (%)	22/25, 88.0
Median stent patency, days (median, min–max)	93 (9–3635)

^§^, *n* (%).

**Table 3:** Survival Analysis: Stent Patency and Overall Survival Outcomes.

Variables	Estimated Mean Period	Standard Deviation	95% CI
Stent patency period, days	278.5	37.1	205.8–351.1
Overall survival, days	191.2	27.4	137.4–245.0

**Table 4:** Incidence of Post-procedural Adverse Events.

Variables ^+^	Results
Cholangitis	2
Stent expansion failure	0
Cholecystitis	0
Pancreatitis	0
bleeding	0

^+^, *n*.

**Table 5:** Predictive Factors for Stent Patency: Univariate and Multivariate Analysis.

Variables	Univariate			Multivariate		
	Odds Ratio	95% CI	*p*-Value *	Odds Ratio	95% CI	*p*-Value *
Age	0.7	0.2–2.3	0.9	0.5	0.1–1.8	0.3
Sex	0.5	0.2–1.4	0.9	0.4	0.1–1.3	0.1
Diagnosis	0.9	0.3–2.7	0.9	1.0	0.3–3.2	1.0
Total bilirubin	0.9	0.3–2.9	0.9	0.6	0.2–2.1	0.4
ALP	2.2	0.3–16.4	0.5	2.6	0.3–21.7	0.4

Abbreviations: ALP, alkaline phosphatase. *, *p* values were calculated using the *t*-test for continuous variables and the Chi-square test for categorical variables.

## Data Availability

The raw data supporting the conclusions of this article will be made available by the authors on request.

## Abbreviations

MHBO, malignant hilar biliary obstruction; SEMS, self-expandable metal stent; ERCP, endoscopic retrograde cholangiopancreatography; PT-INR, prothrombin time-international normalized ratio; ALP, alkaline phosphatase; OS, overall survival; IRB, institutional review board; CT, computed tomography; MRI, magnetic resonance imaging; CI, confidence interval; HR, hazard ratio.
